# Enteric glial cells as regulators of the gut-brain axis: hormonal signaling, stress responses and progenitor potential

**DOI:** 10.3389/fnins.2026.1922802

**Published:** 2026-08-11

**Authors:** Sarah Stahlke, Felix Lappe, Veronika Matschke, Carsten Theiss

**Affiliations:** 1 Department of Cytology, Faculty of Medicine, Ruhr-University Bochum, Bochum, Germany; 2 Medical Imaging Center (MIC), Electron Microscopy Medical Analysis – Core Facility (EMMACF), Faculty of Medicine, Ruhr-University Bochum, Bochum, Germany; 3 International Graduate School of Neuroscience (IGSN), Ruhr-University Bochum, 44801 Bochum, Germany

**Keywords:** enteric glia progenitors, enteric glial cells, enteric glial differentiation, gut-brain axis, stress-response

## Abstract

Enteric glial cells (EGCs) are increasingly recognized as key regulators of the gut-brain axis, extending far beyond their traditional role as structural support cells within the enteric nervous system. Growing evidence demonstrates that EGCs integrate neuronal, immune, epithelial, and microbial signals to maintain intestinal homeostasis and coordinate adaptive responses to physiological and pathological stimuli. This review summarizes current knowledge on enteric glial heterogeneity, stress responsiveness, hormonal regulation, and their emerging capacity for regenerative plasticity. While inflammatory and oxidative stress responses of EGCs are increasingly well characterized, their regulation by endocrine signaling remains poorly understood despite accumulating evidence for sex-dependent differences and hormone receptor expression. Furthermore, recent studies suggest that EGCs retain facultative progenitor properties and may contribute to tissue repair and neuronal regeneration under specific conditions. By integrating these largely independent research areas, we propose a conceptual framework in which enteric glia function as central signal integrators linking stress, hormonal signaling, and regenerative responses within the gut-brain axis. A better understanding of these mechanisms may identify enteric glia as promising therapeutic targets for gastrointestinal and neurodegenerative diseases.

## 1 Introduction

Enteric glial cells (EGCs) are increasingly recognized as active regulators of gastrointestinal physiology and essential components of the gut-brain axis (GBA). Traditionally regarded as supportive cells of the enteric nervous system (ENS), EGCs are now known to participate in a broad range of functions, including the maintenance of intestinal epithelial barrier integrity, modulation of immune responses, regulation of neurotransmission, and bidirectional communication with the gut microbiota (Neunlist et al., 2014; Grubišić and Gulbransen, 2016). Through these diverse activities, EGCs integrate neural, immune, microbial, and endocrine signals and thereby contribute to the maintenance of intestinal homeostasis and gut-brain communication.

Growing evidence suggests that enteric glia are critically involved in disorders associated with GBA dysfunction. Alterations in EGC activity have been described in inflammatory bowel disease, irritable bowel syndrome, and neurodegenerative disorders such as Parkinson’s disease. In Parkinson’s disease, reactive EGCs have been implicated in the regulation of intestinal motility, visceral sensory signaling, and immune polarization, highlighting their potential contribution to both local gastrointestinal pathology and systemic neuroimmune processes (Thomasi et al., 2023). Despite these advances, the mechanisms by which EGCs integrate environmental and systemic signals to influence gut-brain communication remain incompletely understood.

One emerging area of research concerns the role of EGCs as stress-responsive cells. Experimental studies have demonstrated that chronic stress alters enteric glial activity and disrupts intestinal homeostasis. In a rat model of chronic water-avoidance stress, pharmacological inhibition of enteric glia reversed stress-induced alterations in microbiota composition and restored the expression of the tight junction proteins occludin and claudin-1 (Lu et al., 2024). Furthermore, EGCs have been proposed to participate in microbiota-brain communication through the sensing of microbial metabolites and the modulation of neuroinflammatory pathways (Blair et al., 2025). These findings suggest that enteric glia may represent an important cellular interface through which environmental stressors influence GBA function. However, current knowledge is derived predominantly from animal models and mechanistic studies, and translation to human physiology remains limited.

In parallel, increasing attention has been directed toward endocrine regulation of the ENS. Steroid hormones and stress-related endocrine pathways are established modulators of gastrointestinal physiology and gut-brain communication, influencing intestinal permeability, immune responses, and neuronal function. Nevertheless, their specific effects on enteric glial biology remain poorly characterized. Emerging evidence suggests that EGCs may respond to hormonal and neuroendocrine signals and thereby contribute to the integration of systemic endocrine cues within the ENS. Understanding these mechanisms may provide important insights into how physiological states such as aging, stress, and hormonal fluctuations influence intestinal and neurological health.

Another rapidly evolving area of research concerns the remarkable cellular plasticity of enteric glia. Although EGCs are generally quiescent under physiological conditions in mammals, accumulating evidence indicates that they retain progenitor-like properties and may contribute to neuronal regeneration following injury. In zebrafish, enteric glia actively proliferates and generates new enteric neurons under homeostatic conditions through mechanisms involving Notch signaling (McCallum et al., 2020). In mammals, lineage-tracing and single-cell transcriptomic studies have demonstrated that subsets of EGCs maintain accessibility at neurogenic loci, supporting their capacity for neuronal differentiation under specific pathological conditions (McCallum et al., 2020; Laddach et al., 2023). These findings position enteric glia as promising candidates for endogenous ENS repair and regeneration (Boesmans et al., 2022; Lefèvre et al., 2023).

Although substantial progress has been made in characterizing enteric glial biology, the fields of hormonal regulation, stress responsiveness, and progenitor potential have largely developed independently. To date, no comprehensive synthesis has integrated these emerging properties of EGCs within the broader framework of gut-brain axis regulation. Yet, accumulating evidence suggests that these processes may be closely interconnected. Hormonal and stress-related signals influence cellular plasticity and neuroimmune function in multiple tissues, raising the possibility that similar mechanisms shape enteric glial behavior and adaptive responses within the ENS.

In this review, we summarize current knowledge on enteric glial cells as regulators of the gut-brain axis, with particular emphasis on hormonal signaling, stress responses, and progenitor potential. We discuss how these interconnected functions may contribute to ENS homeostasis, adaptation, and disease and highlight key challenges and future directions for understanding the role of enteric glia in gut-brain axis physiology.

## 2 Cellular diversity and functional heterogeneity of enteric glial cells

**FIGURE 1:**
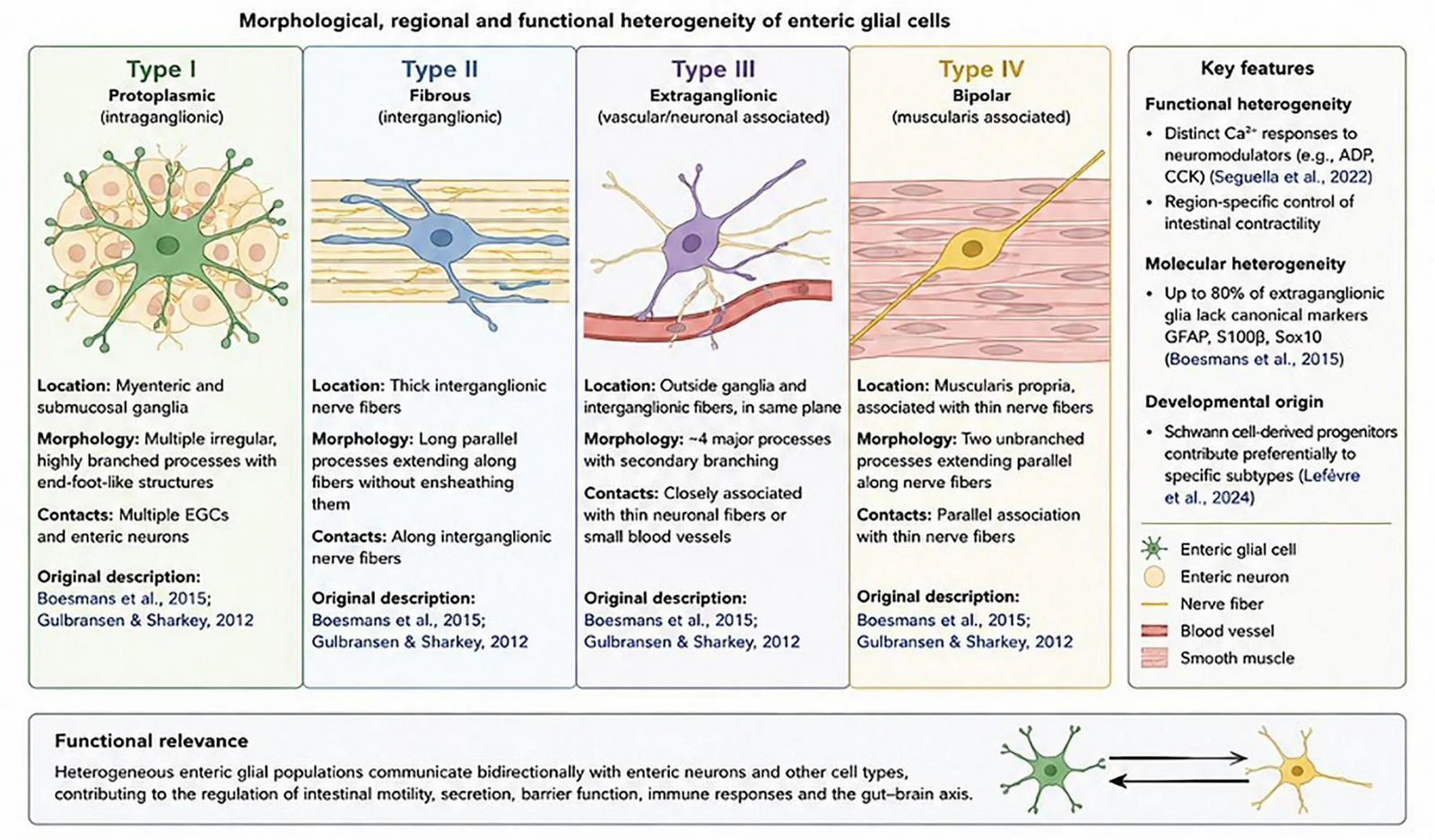
Topo-morphological and functional heterogeneity of enteric glial cells. Four major EGC subtypes have been described in the mouse ENS, differing in morphology, location and molecular/function profiles. ADP, adenosine diphosphate; CCK, cholecystokinin; ENS, enteric nervous system; EGC, enteric glial cells. Figure generated with assistance of ChatGPT (OpenAI, GPT-5.3-mini).

Enteric glial cells (EGCs) exhibit marked morphological, regional, and functional heterogeneity within the ENS (Table 1). Whether this heterogeneity reflects stable cellular subtypes or dynamic functional states remains a matter of ongoing debate (Valès et al., 2018). Based on combined morphological, molecular, and functional analyses, four major EGC subtypes can be distinguished (Figure 1).

**TABLE 1:** Enteric glial heterogeneity across functional levels.

*Level*	Evidence	Key study
*Morphological heterogeneity*	Type I-IV glia	Boesmans et al., 2015
*Functional heterogeneity*	Distinct Ca^2+^ responses	Seguella et al., 2022
*Molecular heterogeneity*	TACR3-positive populations	Tachykinin study 2025/26
*Developmental heterogeneity*	Schwann cell-derived subsets	Lefèvre et al., 2023
*Stem-cell niche specialization*	Distinct niche-regulating populations	Baghdadi et al., 2022
*Dynamic state transitions*	Diversity vs plasticity concept	Valès et al., 2018

Type I (protoplasmic, intraganglionic glia) are located within myenteric and submucosal ganglia. They are characterized by multiple highly branched processes with end-foot-like structures and establish extensive contacts with enteric neurons and neighboring glial cells. These cells are considered highly integrated within intraganglionic signaling networks.

Type II (fibrous, interganglionic glia) are primarily located along thick interganglionic nerve fiber tracts. They exhibit elongated, parallel processes that extend along nerve fibers without fully ensheathing them, suggesting a role in long-range structural and signaling support within enteric plexuses.

Type III (extraganglionic, vascular/neuronal-associated glia) are positioned outside ganglia and interganglionic fiber bundles but within the same tissue plane. They display several major processes with secondary branching and are closely associated with small nerve fibers and blood vessels, indicating potential involvement in neurovascular and metabolic coupling.

Type IV (bipolar, muscularis-associated glia) are located in the muscularis propria and are associated with thin nerve fibers. They typically exhibit two unbranched processes extending parallel to nerve fibers, suggesting a specialized role in neuromuscular regulation.

Functional studies indicate that these EGC subtypes display distinct calcium signaling profiles in response to neuromodulators such as adenosine diphosphate (ADP) and cholecystokinin (CCK), supporting functional heterogeneity across intestinal regions (Seguella et al., 2022). Recent transcriptomic analyses have further refined, and in part challenged, the concept of discrete enteric glial subtypes. A comprehensive single-cell study identified transcriptionally distinct mucosal and muscularis glial populations and demonstrated that TACR3 marks a specialized subset of enteric glia responsive to Neurokinin-B signaling. Tachykinin signaling was further shown to actively regulate glial diversification, indicating that enteric glial heterogeneity is not solely developmental in origin but remains dynamically regulated throughout life, and may better be described as a continuous transcriptional landscape rather than a set of discrete, fixed subtypes (Muppirala et al., 2026). Consistent with this view, molecular analyses show that a substantial fraction of extraganglionic glia lack canonical markers such as glial fibrillary acidic protein (GFAP), S100β, and Sox10, pointing to dynamic transcriptional regulation rather than fixed lineage identity (Boesmans et al., 2014). Developmental studies further demonstrate that Schwann cell-derived progenitors contribute differentially to specific glial subtypes during postnatal ENS maturation (Lefèvre et al., 2023).

Despite the extensive characterization, the functional consequences of enteric glial heterogeneity remain only partially understood. A plethora of functions have been described or postulated for subtypes I–III, including the regulation of oxidative stress (Abdo et al., 2010), neuroinflammation (Ibiza et al., 2016), neurogenesis (Laranjeira et al., 2011; Belkind-Gerson et al., 2017), and gliogenesis (Rao and Gershon, 2015), whereas the functions of some Type III subpopulations and Type IV cells have received comparatively little attention.

Collectively, these findings support the concept that EGCs represent a highly heterogeneous and dynamically regulated cellular network with region-specific specialization and functional diversity within the ENS, though the underlying classification remains incompletely resolved. In this regard, it should be emphasized that the four-subtype classification introduced above is based primarily on morphological and topographical criteria and should not be interpreted as a definitive molecular taxonomy; the boundaries between this morphological framework and the continuous transcriptional heterogeneity described above remain to be reconciled and warrant further investigation.

## 3 Enteric glia as integrators of gut-brain axis signaling

EGCs are increasingly recognized as key integrators of GBA signaling. Rather than functioning solely as structural support cells within the ENS, EGCs actively coordinate communication between enteric neurons, epithelial cells, immune cells, and the intestinal microbiota. Through these interactions, they contribute to the integration of neural, immune, microbial, and endocrine signals that regulate gastrointestinal homeostasis.

A central function of EGCs is their bidirectional communication with enteric neurons. Glial signaling influences neuronal excitability, synaptic activity, and gastrointestinal reflex circuits, thereby contributing to the regulation of intestinal motility, secretion, and sensory processing (Grubišić and Gulbransen, 2016; Seguella and Gulbransen, 2021). Active neuron-glia communication has emerged as a critical mechanism underlying regional specialization and adaptive responses within the ENS.

In addition to their interactions with neurons, EGCs are important regulators of intestinal immune homeostasis. Bidirectional signaling between enteric glia and immune cells modulates inflammatory responses and contributes to the maintenance of mucosal integrity (Seguella and Gulbransen, 2021). EGCs also communicate with epithelial cells through the release of soluble mediators that influence epithelial barrier function, thereby regulating intestinal permeability and host defense mechanisms (Sharkey, 2015). Among microbial metabolites, short-chain fatty acids (SCFAs) have the most direct evidence of EGC involvement: butyrate, propionate, and acetate directly modulate the secretion of neurotrophic factors (e.g., GDNF, TGF-β1) and pro-/anti-inflammatory cytokines by EGCs via G-protein-coupled receptor signaling and histone deacetylase inhibition, thereby influencing epithelial barrier integrity. In contrast, although bile acids and tryptophan-derived indole metabolites are established regulators of other gut-brain axis components, including enteroendocrine cells and central microglia, their direct effects on enteric glial biology have not yet been investigated and represent an important gap for future research.

The integrative role of EGCs extends beyond local gastrointestinal physiology and has important implications for disease. Increasing evidence suggests that enteric glia participate in neuroinflammatory processes associated with inflammatory bowel disease and neurodegenerative disorders, including Parkinson’s disease (D’Antongiovanni et al., 2023). Furthermore, EGCs have been implicated in the development and maintenance of chronic visceral pain through their interactions with neuronal and immune signaling pathways (Morales-Soto and Gulbransen, 2019).

Taken together, these findings support the concept that EGCs function as central cellular hubs within the gut-brain axis. By integrating signals from neuronal, immune, epithelial, microbial, and potentially endocrine sources, enteric glia contribute to the dynamic regulation of intestinal homeostasis and represent key mediators linking gastrointestinal and neurological function. It should be noted that much of this evidence derives from correlative observations in disease models rather than from mechanistic loss-of-function studies in EGCs specifically; consequently, it often remains unclear whether glial changes are a cause or a consequence of neuroimmune dysregulation.

## 4 Enteric glia as stress-responsive cells

EGCs are increasingly recognized as highly dynamic stress-responsive cells that actively adapt to changes in the intestinal microenvironment. In response to inflammatory, oxidative, and psychological stressors, EGCs undergo phenotypic and functional alterations that influence neuronal survival, epithelial barrier integrity, immune activation, and gut-brain axis signaling. Although the underlying mechanisms remain incompletely understood and most evidence originates from experimental models, current data suggest that EGCs represent central cellular mediators of stress adaptation within the ENS (Table 2).

**TABLE 2:** Stress-induced responses of enteric glial cells.

*Stress type*	EGC response	Molecular markers/pathways	Functional consequences	References
*Inflammatory stress*	Reactive gliosis	↑ GFAP	Glial activation and immune cell recruitment	von Boyen et al., 2004
*Inflammatory stress*	Reactive gliosis	GFAP-positive myenteric glia	Response to acute intestinal inflammation	Rosenbaum et al., 2016
*Inflammatory stress*	Reactive gliosis	↑ GFAP	Altered GI homeostasis in endometriosis model	Rivera-Arce et al., 2024
*Inflammatory stress*	Pro-inflammatory phenotype	S100B-RAGE-NFκB signaling	Neuronal loss, mucosal injury, inflammation	Costa et al., 2019
*Inflammatory stress*	Cytokine secretion	IL-1β, TNF-α, LPS responsiveness	Sustained neuroinflammation	Neunlist et al., 2014; Linan-Rico et al., 2023
*Oxidative stress*	Neuroprotective response	Glutathione release	Protection of enteric neurons from ROS-induced apoptosis	Abdo et al., 2010
*Oxidative stress*	Cellular injury	RAC1, NADPH oxidase, p66SHC	Oxidative damage of EGCs under hyperglycemia	Jiang et al., 2024
*Oxidative stress*	Phenotypic switch	Excessive ROS	Shift toward pro-inflammatory glial phenotype	Almeida et al., 2022
*Psychological stress*	Barrier regulation	NOS signaling, cholinergic pathways	Dysbiosis and barrier dysfunction	Lu et al., 2024
*Psychological stress*	Microbiota modulation	EGC-dependent mechanisms	Changes in microbiota composition	Lu et al., 2024
*Obesity-associated stress*	ENS dysfunction	Oxidative-inflammatory pathways	Impaired gut homeostasis	Bellitto et al., 2026

Inflammatory Stress: Inflammation is among the best-characterized activators of enteric glial responses. Similar to reactive astrocytes in the central nervous system, EGCs undergo reactive gliosis characterized by increased expression of glial markers, altered secretory profiles, and enhanced interactions with immune cells. Pro-inflammatory mediators such as interleukin-1β (IL-1β), tumor necrosis factor-α (TNF-α), and lipopolysaccharide (LPS) induce upregulation of GFAP in cultured rat EGCs, demonstrating a direct responsiveness of enteric glia to inflammatory stimuli (von Boyen et al., 2004). Subsequent studies have identified specific glial populations that are particularly sensitive to inflammatory challenges. In vivo, GFAP-expressing myenteric glia exhibit rapid activation during acute intestinal inflammation and systemic inflammatory insults (Rosenbaum et al., 2016). Comparable findings have been reported in experimental models of endometriosis, where increased GFAP expression in the ileum was associated with intestinal inflammation and reactive gliosis (Rivera-Arce et al., 2024). This model is of particular relevance because endometriosis is frequently accompanied by IBS-like gastrointestinal symptoms and chronic pelvic inflammation, illustrating that reactive gliosis is not restricted to classical intestinal inflammatory contexts such as IBD or infection, but represents a shared cellular response of EGCs across diverse, even extra-intestinal, inflammatory conditions affecting the gut.

A further hallmark of inflammatory EGC activation is the increased production of S100B. In a mouse model of 5-fluorouracil-induced intestinal mucositis, EGC-derived S100B contributed to enteric neuronal loss, inflammatory signaling, oxidative stress, and tissue damage through activation of the RAGE/NF-κB pathway (Costa et al., 2019). Together with GFAP upregulation, elevated S100B expression represents a characteristic feature of reactive gliosis, paralleling well-established responses of astrocytes in the CNS (Pekny et al., 2014). These findings collectively indicate that EGCs are active participants in enteric neuroinflammation rather than passive bystanders. Through cytokine secretion, modulation of immune-cell recruitment, and regulation of neuronal survival, enteric glia contribute to both the initiation and progression of inflammatory responses within the gastrointestinal tract (Neunlist et al., 2014; Linan-Rico et al., 2023).

Direct comparison between these studies, however, is complicated by considerable methodological heterogeneity. Current knowledge derives largely from rodent models employing distinct inflammatory paradigms, including lipopolysaccharide administration, chemically induced intestinal inflammation, endometriosis, and chemotherapy-induced mucositis, while human mechanistic data addressing these questions remain lacking. Consequently, increased GFAP or S100B expression is best interpreted as a general marker of reactive gliosis rather than a disease-specific glial phenotype, and reactive enteric gliosis itself should not be regarded as uniformly protective or detrimental. While glial-derived antioxidant and neurotrophic support, such as glutathione release (Abdo et al., 2010) and BDNF-mediated inhibition of glial apoptosis (Steinkamp et al., 2012), can limit neuronal injury, the same reactive phenotype can amplify local inflammation through S100B-RAGE-NF-κB signaling and cytokine release, contributing to neuronal loss and chronic visceral hypersensitivity (Costa et al., 2019; Morales-Soto and Gulbransen, 2019). The net outcome likely depends on the intensity and duration of the initiating stimulus and on glial subtype-specific responses that remain incompletely characterized.

Oxidative Stress: In addition to inflammatory stimuli, EGCs are highly responsive to oxidative stress. Reactive oxygen species (ROS) are generated during inflammation, metabolic dysfunction, aging, and psychological stress and represent important modulators of ENS homeostasis. A protective role of EGCs against oxidative injury was demonstrated by Abdo et al. (2010), who showed that cultured rat EGCs protect enteric neurons from dopamine- and hydrogen peroxide-induced oxidative damage through the release of reduced glutathione, significantly reducing neuronal apoptosis and improving neuronal survival under stress conditions. A related, though less well-characterized, protective mechanism involves the Nrf2 antioxidant transcription pathway, a master regulator of cellular redox defense in other glial and epithelial cell types. EGCs have been identified as a source of 15-deoxy-Δ12,14-prostaglandin J2 (15d-PGJ2), a lipid mediator capable of activating Nrf2-dependent antioxidant gene expression (e.g., NQO1, HO-1) and contributing to neuroprotection against oxidative stress (Abdo et al., 2010). Direct evidence for Nrf2 activity within EGCs, however, remains limited to this single study, and its functional relevance for glial redox homeostasis under physiological and pathological conditions warrants further investigation.

However, EGCs themselves are vulnerable to excessive oxidative stress. Hyperglycemic conditions induce oxidative injury in cultured EGCs through activation of redoxosome-associated signaling pathways involving RAC1, NADPH oxidase, and p66SHC (Jiang et al., 2024). Pharmacological inhibition of these pathways effectively attenuated cellular damage, highlighting their importance in stress-induced glial dysfunction. Beyond direct cellular injury, oxidative stress influences glial phenotype and neuron-glia communication. Alterations in EGC morphology, density, and neurochemical profiles have been observed under conditions of increased oxidative burden, while excessive ROS promotes a shift toward pro-inflammatory glial states that may impair ENS homeostasis (Almeida et al., 2022). More recently, obesity-associated oxidative stress has been linked to enteric dysfunction, further emphasizing the importance of redox regulation within the ENS. Mechanistically, diets rich in lipids and carbohydrates are proposed to promote intestinal dysbiosis and epithelial barrier failure, while obesity-associated hyperglycemia, hyperlipidemia, and micronutrient deficiencies further enhance local ROS generation. Under these conditions, EGCs are thought to switch toward a reactive, pro-inflammatory phenotype that disrupts neuron-glia communication and amplifies both local and systemic inflammatory signaling, underscoring the potential therapeutic value of antioxidant interventions (Bellitto et al., 2026).

Taken together, current evidence suggests that EGCs occupy a dual role during oxidative stress, acting as neuroprotective regulators that buffer oxidative injury while themselves becoming dysfunctional once antioxidant defense mechanisms are overwhelmed. Important differences exist between experimental models. Acute oxidative injury predominantly reveals the neuroprotective capacity of EGCs through glutathione and Nrf2-related pathways, whereas chronic metabolic stress, as in obesity or hyperglycemia, is associated with sustained activation of intracellular redox pathways and a shift toward reactive phenotypic changes. These findings appear complementary rather than contradictory, reflecting distinct stages or intensities of oxidative challenge rather than opposing mechanisms. Nevertheless, most available evidence originates from rodent models or immortalized cell lines, and corresponding human data remain largely absent.

Neuroendocrine Stress: Compared with inflammatory and oxidative stress, considerably less is known about the direct effects of neuroendocrine stress on enteric glial biology. While activation of the hypothalamic-pituitary-adrenal (HPA) axis and glucocorticoid signaling are recognized as major regulators of gut-brain axis function, direct evidence linking these pathways to EGC-specific responses remains scarce.

One of the few studies addressing this question demonstrated that chronic water-avoidance stress alters EGC activity and induces changes in intestinal microbiota composition, epithelial barrier integrity, and neuronal signaling (Lu et al., 2024). Pharmacological inhibition of enteric glia reversed stress-induced dysbiosis and restored the expression of the tight junction proteins occludin and claudin-1, indicating that EGCs are essential mediators of stress-induced intestinal dysfunction. Mechanistically, these effects were associated with alterations in nitric oxide synthase activity and cholinergic neuronal signaling.

Neuroendocrine stress therefore remains one of the least investigated aspects of enteric glial biology. Current conclusions rely almost exclusively on this single chronic stress model in rats, making it difficult to distinguish direct endocrine effects from secondary consequences of altered microbiota composition, immune activation, or barrier dysfunction. No studies to date have systematically compared acute and chronic stress paradigms, directly investigated glucocorticoid signaling or HPA-axis regulation in EGCs, or examined these mechanisms in human tissue. Consequently, the role of EGCs in neuroendocrine stress should currently be regarded as an important knowledge gap rather than an established biological mechanism, and the findings of Lu et al. (2024) should be interpreted with corresponding caution until independently replicated. Collectively, current evidence indicates that enteric glia exhibit context-dependent responses to different forms of cellular stress, with inflammatory stress representing the most extensively characterized stimulus across multiple experimental models, and considerably less known about oxidative and, in particular, neuroendocrine stress. Direct comparability across these stress types remains limited by substantial heterogeneity in stressor type, duration, and species. Most inflammatory and oxidative stress data derive from acute, high-dose challenges in rodent primary cultures (e.g., LPS, TNF-α, hydrogen peroxide), which may not reflect the low-grade, chronic stress typically implicated in human gastrointestinal disease, whereas the single available neuroendocrine-stress study (Lu et al., 2024) instead used a chronic *in vivo* paradigm, precluding direct comparison with these acute *in vitro* models. Such differences in experimental design likely contribute to the apparent divergence between protective (e.g., glutathione- and Nrf2-mediated) and detrimental (e.g., S100B-RAGE-NF-κB-mediated) glial responses reported across studies and should be considered when extrapolating findings across stress types. Future investigations should therefore employ standardized experimental approaches and include human tissue to clarify whether distinct stressors induce unique glial phenotypes or instead represent different manifestations of a common adaptive response. Taken together, these adaptive mechanisms suggest that EGCs contribute importantly to ENS homeostasis under physiological and pathological stress conditions; it should be noted, however, that this conclusion is derived predominantly from preclinical and largely indirect evidence, and direct confirmation in human tissue remains limited.

## 5 Hormonal regulation of enteric glial function

Compared with the growing body of literature addressing inflammatory and oxidative stress responses in enteric glia, remarkably little is known about the direct hormonal regulation of EGC function. Nevertheless, accumulating evidence suggests that endocrine signaling represents an important but underexplored component of enteric glial biology. Sex-specific differences observed in gastrointestinal disorders, neurodegenerative diseases, and gut-brain axis dysfunction strongly suggest that hormonal signaling contributes to ENS regulation. However, whether EGCs serve merely as downstream responders or act as direct targets of hormonal signaling remains largely unresolved.

Steroid Hormones: Among endocrine regulators, estrogens have received the greatest attention within the broader context of gut-brain axis physiology. Estrogen signaling influences intestinal permeability, immune responses, visceral sensitivity, and microbiota composition. In a spontaneous rat model of disorders of gut-brain interaction, estrogen was shown to directly contribute to colonic hypersensitivity in both males and females (Accarie et al., 2022). Furthermore, sex-dependent differences in gastrointestinal disorders such as irritable bowel syndrome have long suggested a role for fluctuating estrogen levels in ENS regulation (Hogan et al., 2009).

Direct evidence linking estrogens to EGC function remains scarce. However, several observations support a potential interaction. Estrogen receptors ERα, ERβ, and GPER have been identified throughout the murine gastrointestinal tract, with receptor expression exhibiting sex-specific patterns (Liu et al., 2019). Furthermore, ERβ signaling promotes proliferation and differentiation of enteric neuronal progenitor cells, enhancing the generation of both enteric neurons and glia *in vitro* (D’Errico et al., 2018). These findings suggest that estrogen signaling may influence enteric glial development, maintenance, or plasticity, although direct mechanistic studies are currently lacking.

Similar knowledge gaps exist regarding progesterone. While the neuroprotective effects of progesterone are well established in the central nervous system, relatively few studies have examined its role within the ENS. Stegemann et al. (2023) demonstrated that enteric neurons express multiple progesterone receptors, including PR-A/B, mPRα, mPRβ, and PGRMC1. In a rotenone-induced model of Parkinson-like neurotoxicity, progesterone significantly reduced neuronal cell death, with PGRMC1 acting as a key mediator of this protective effect. Likewise, Jarras et al. (2019) reported neuroprotective and anti-inflammatory actions of progesterone in the myenteric plexus of MPTP-treated mice. More recently, Kallabis et al. (2026) identified progesterone receptor expression throughout the human gastrointestinal tract and demonstrated receptor localization not only in neurons but also in Sox10- and GFAP-positive enteric glial cells. Importantly, receptor expression differed between males and females, providing some of the strongest evidence to date for direct progesterone responsiveness of human EGCs.

Compared with estrogens and progesterone, even less is known about androgen signaling in enteric glia. Nevertheless, sexual dimorphism of enteric glial phenotypes has been reported, and early-life stress has been shown to induce sex-specific glial alterations in experimental models (Gonzales et al., 2024). While androgen receptor signaling clearly regulates gastrointestinal motility and ENS function, current evidence suggests that these effects are primarily mediated through enteric neurons rather than glia (Rastelli et al., 2022). Whether androgens directly influence enteric glial physiology remains unknown.

Neurotrophic Signaling: Beyond classical steroid hormones, enteric glia both respond to and produce neurotrophic factors that are essential for ENS maintenance and repair. The physiological importance of glial-derived trophic support is highlighted by studies demonstrating that ablation of GFAP-positive enteric glia results in severe intestinal pathology and loss of gastrointestinal homeostasis (von Boyen and Steinkamp, 2006).

Primary cultures of rat EGCs secrete biologically relevant concentrations of nerve growth factor (NGF), brain-derived neurotrophic factor (BDNF), and glial cell line-derived neurotrophic factor (GDNF), all of which promote neuronal survival and neurite outgrowth (Hansebout et al., 2012). In the developing human ENS, neurotrophin receptors TrkA and TrkB are expressed in both enteric neurons and glia, whereas TrkC expression appears restricted to neurons (Hoehner et al., 1996). Furthermore, BDNF has been shown to inhibit apoptosis of enteric glia during intestinal inflammation, suggesting a protective role in maintaining ENS integrity under pathological conditions (Steinkamp et al., 2012). Similarly, inflammatory stimuli increase NGF secretion from EGCs, linking neurotrophic signaling to inflammatory adaptation and visceral sensitivity (von Boyen and Steinkamp, 2006).

Taken together, current evidence supports the concept that enteric glia participate in endocrine and neurotrophic signaling networks that influence ENS homeostasis, stress adaptation, and potentially regenerative responses (summarized in Table 3). However, direct mechanistic studies remain surprisingly limited. Most available data derive from neuronal analyses, animal models, or indirect observations, whereas investigations specifically targeting EGCs are rare. Consequently, it remains unclear whether hormonal signaling directly regulates enteric glial phenotype, stress responsiveness, and progenitor potential or whether these effects occur secondarily through neuronal or immune pathways.

**TABLE 3:** Hormonal regulation of enteric glial cells.

*Hormonal system*	Evidence in EGCs	Main findings	Key references
*Estrogens (ERα, ERβ, GPER)*	Indirect- Animal studies, *in vitro* studies	Sex differences in ENS-associated diseases; estrogen receptors present throughout the GI tract; ERβ promotes enteric progenitor differentiation into neurons and glia	Hogan et al., 2009; Liu et al., 2019; D’Errico et al., 2018
*Progesterone*	Direct- Animal studies and human tissue analyses	Progesterone receptors identified in enteric neurons and glia; neuroprotection in rotenone and MPTP models; sex-dependent receptor expression	Jarras et al., 2019; Stegemann et al., 2023; Kallabis et al., 2026
*Androgens*	Mostly indirect - Human and animal studies	Sexual dimorphism of EGCs; androgen signaling regulates GI function primarily via neurons; direct glial effects unknown	Rastelli et al., 2022; Gonzales et al., 2024
*Glucocorticoids*	Largely unexplored	No direct mechanistic studies on EGC-specific glucocorticoid signaling identified	Current review
*HPA-axis related hormones*	Largely unexplored - Animal studies	EGCs implicated in stress-induced intestinal dysfunction but direct endocrine regulation unknown	Lu et al., 2024
*Neurotrophic factors (BDNF, NGF, GDNF)*	Direct - *In vitro*, animal, human developmental studies	EGCs both secrete and respond to neurotrophic factors; support neuronal survival and glial homeostasis	Hansebout et al., 2012; Hoehner et al., 1996; Steinkamp et al., 2012; von Boyen et al., 2006

Addressing these questions represents an important challenge for future research. Given the established influence of hormones on neuroinflammation, cellular plasticity, and regeneration in other nervous systems, hormonal regulation of enteric glia may represent a previously underappreciated mechanism linking sex, aging, stress, and GBA function.

## 6 Enteric glia as facultative progenitor cells

Among the most intriguing properties of EGCs is their capacity to act as facultative progenitor cells. Although traditionally viewed as terminally differentiated support cells, accumulating evidence suggests that subsets of EGCs retain developmental programs that enable neuronal differentiation under specific physiological or pathological conditions.

Early studies identified EGCs as potential neuronal and glial progenitors within the ENS. Neunlist et al. (2014) described enteric glia as a cellular population capable of generating both neuronal and glial lineages, while Bondurand et al. (2003) demonstrated that progenitor cells isolated from fetal and postnatal mouse intestine could generate mature neurons and glia and successfully colonize aganglionic gut tissue. More recently, mechanistic insights from lineage-tracing and single-cell sequencing studies have revealed that EGCs maintain accessibility at key neurogenic loci despite their differentiated phenotype. This permissive chromatin landscape may allow glial cells to re-enter developmental programs and generate neurons in response to specific stimuli (Laddach et al., 2023). Beyond their neurogenic potential, recent single-cell analyses suggest that enteric glial populations actively regulate intestinal stem cell niches. Baghdadi et al. (2022) demonstrated that transcriptionally distinct EGC populations occupy specific microenvironments and contribute to local niche signaling, further supporting the concept that enteric glia participate in tissue maintenance and regeneration beyond their classical neuro-supportive functions.

However, the neurogenic capacity of EGCs remains highly context dependent. As emphasized by Gulbransen and Sharkey (2012), enteric glia appear capable of generating neurons only under restricted conditions. Likewise, Gershon (2011) noted that although enteric glia can function as neuronal precursors *in vitro* and under experimental conditions in vivo, they do not constitutively replace neurons during normal tissue homeostasis. Consequently, spontaneous neurogenesis appears to be limited in the healthy mammalian ENS.

Interestingly, substantial species differences have been reported. In zebrafish, enteric glia actively proliferate and continuously generate new enteric neurons under physiological conditions through Notch-dependent mechanisms (McCallum et al., 2020). In contrast, mammalian EGCs generally remain quiescent and require injury, inflammation, or experimental manipulation to activate neurogenic programs. These findings suggest that the regenerative potential of enteric glia is evolutionarily conserved but differentially regulated across species.

Importantly, the emerging concepts discussed in the previous chapters raise the possibility that neurogenic plasticity is not an isolated property of EGCs but may be closely linked to their stress-responsive and hormone-sensitive nature. In other nervous systems, inflammatory mediators, steroid hormones, and growth factors are well-established regulators of stem cell activity and cellular plasticity. Whether similar mechanisms govern the activation of neurogenic programs in enteric glia remains largely unknown. Understanding how stress- and hormone-dependent signaling pathways influence EGC plasticity may therefore represent an important step toward harnessing endogenous regenerative mechanisms within the ENS.

Translational Limitations: From Rodent Models to Human ENS: The vast majority of findings summarized in this review derive from mouse, rat, or zebrafish models. Direct human data remain scarce and are largely limited to receptor-expression studies (e.g., Kallabis et al., 2026) or descriptive immunohistochemical analyses. Important species differences further complicate translation: for example, GFAP expression is a near-universal marker of rodent enteric glia but is only detectable in a subset of glial cells in the healthy human gut, becoming more prominent under pathological conditions (Le Berre-Scoul et al., cited via Enteric Glia: S100, GFAP, and Beyond). Access to viable human enteric tissue is additionally constrained by the invasiveness of full-thickness biopsies, limiting functional and mechanistic studies to surgical specimens, organoid models, or post-mortem tissue. These constraints should be considered when interpreting the mechanistic conclusions drawn from animal studies discussed throughout this review.”

## 7 A unified model: linking hormones, stress and regenerative plasticity

**FIGURE 2:**
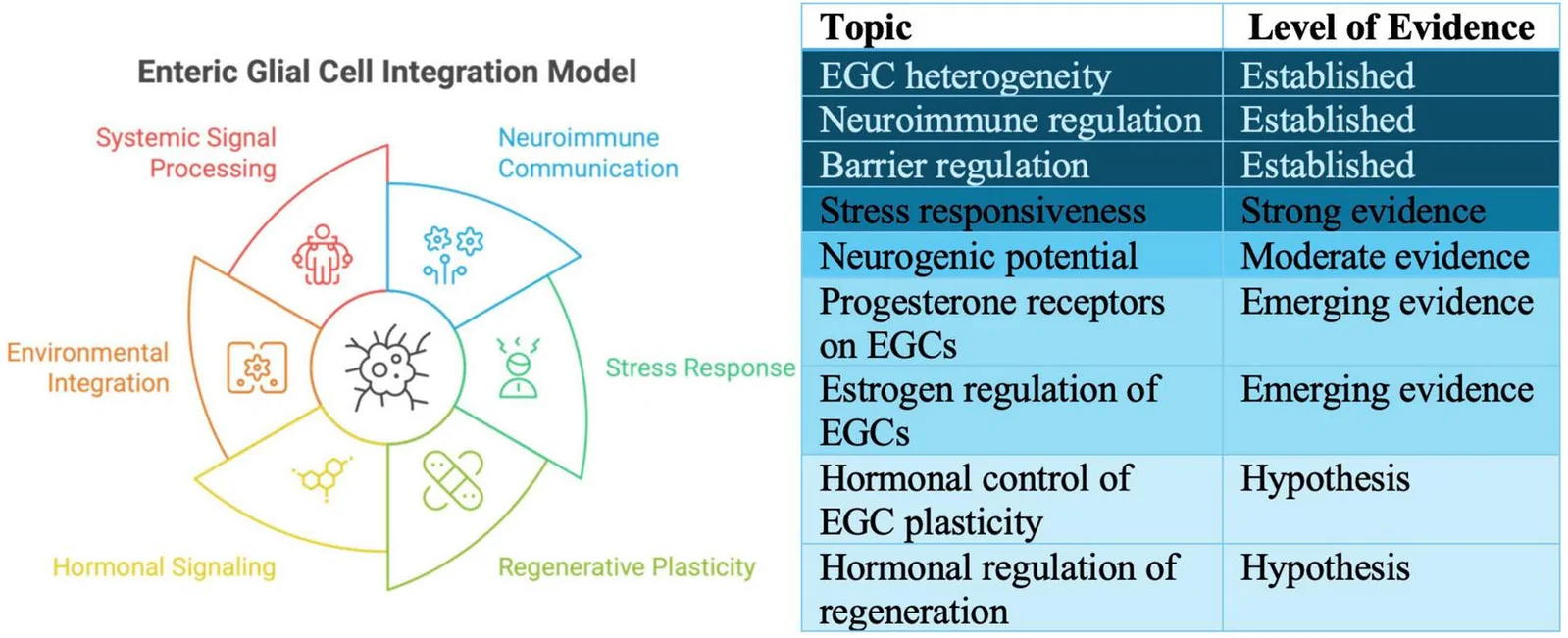
Graphic summary of the key factors that interact with enteric glial cells. Generated with napkin.AI.

It is important to distinguish between the directly established, the emerging, and the largely hypothetical aspects of enteric glial biology discussed in this review. While stress-induced reactive gliosis is comparatively well documented, hormonal regulation of EGCs remains supported by only a small number of direct studies, and the integrative model proposed below is therefore a conceptual hypothesis rather than an established mechanism. The studies discussed throughout this review suggest that enteric glial cells occupy a unique position within the gut-brain axis, acting simultaneously as regulators of neuroimmune communication, stress-responsive cells, and facultative progenitors (Figure 2). Although these functions have traditionally been investigated separately, accumulating evidence indicates that they may represent interconnected aspects of enteric glial biology.

Inflammatory and oxidative stress induce profound changes in enteric glial phenotype, including reactive gliosis, cytokine secretion, altered neurotrophic support, and modulation of epithelial barrier function. At the same time, hormonal signaling pathways influence neuroinflammatory responses, cellular survival, and tissue plasticity throughout the nervous system. Emerging evidence further suggests that enteric glia express hormone receptors and respond to endocrine signals, although direct mechanistic studies remain scarce.

The convergence of these pathways raises the possibility that enteric glia function as central integrators of environmental and systemic signals within the ENS. Under physiological conditions, EGCs contribute to neuronal maintenance and gastrointestinal homeostasis. Following injury or stress, however, activation of inflammatory, endocrine, and neurotrophic pathways may shift glial cells toward adaptive phenotypes that support tissue repair and, under specific circumstances, neuronal regeneration.

We therefore propose a conceptual framework in which hormonal signaling and stress-related pathways act as upstream regulators of enteric glial plasticity. In this model, EGCs integrate signals derived from the immune system, intestinal microbiota, and endocrine organs and translate them into coordinated responses affecting neuronal survival, barrier integrity, and regenerative capacity. While substantial experimental work will be required to validate this hypothesis, such a framework may help explain how systemic physiological states influence ENS adaptation and gut-brain axis function across the lifespan.

## 8 Future perspectives and therapeutic opportunities

Despite substantial advances in enteric glial biology, major knowledge gaps (see Table 4) remain regarding the mechanisms by which hormones, stress-related signaling pathways, and regenerative programs interact within EGCs. Future studies should prioritize the identification of hormone receptor expression patterns across distinct glial subpopulations and determine whether endocrine signaling directly regulates enteric glial phenotype, neuroimmune function, and neurogenic potential.

**TABLE 4:** Major knowledge gaps and future research priorities.

Topic	Current evidence	Major gap
Estrogen-EGC signaling	Indirect	No direct mechanistic studies
Progesterone-EGC signaling	Emerging	Functional consequences unknown
Androgen-EGC signaling	Very limited	Direct receptor-mediated effects unknown
HPA-axis regulation of EGCs	Sparse	Virtually unexplored
Hormonal regulation of EGC progenitor function	None	Major gap
Hormonal regulation of EGC stress responses	None	Major gap
Human EGC heterogeneity	Emerging	Lack of spatial and functional characterization
EGC regenerative activation	Experimental	Translation to humans unclear

Recent developments in single-cell transcriptomics, spatial transcriptomics, lineage tracing, and organoid-based ENS models provide powerful tools to address these questions. In particular, integrating these approaches with experimental models of stress, aging, and neurodegeneration may reveal previously unrecognized mechanisms governing enteric glial plasticity.

From a translational perspective, enteric glia represent attractive therapeutic targets (Table 5). Their accessibility within the gastrointestinal tract, combined with their roles in barrier maintenance, immune regulation, neuronal support, and tissue repair, positions them as potential mediators of interventions aimed at restoring gut-brain axis homeostasis. Pharmacological modulation of glial signaling pathways, neurotrophic support mechanisms, or hormone-responsive pathways may therefore offer novel therapeutic opportunities for gastrointestinal disorders, neurodegenerative diseases, and age-associated ENS dysfunction.

**TABLE 5:** Potential therapeutic targets in enteric glial cells.

*Target*	Mechanism	Potential therapeutic application	Current evidence
*Progesterone-PGRMC1 signaling*	Neuroprotection, anti-apoptotic signaling	Parkinson’s disease, ENS degeneration	Stegemann et al., 2023
*Progesterone receptor pathways*	Anti-inflammatory signaling	Neurodegenerative diseases, enteric inflammation	Jarras et al., 2019
*S100B-RAGE axis*	Modulation of reactive gliosis	Mucositis, intestinal inflammation	Costa et al., 2019
*Redoxosome/p66SHC signaling*	Reduction of oxidative stress	Diabetic enteropathy, metabolic disorders	Jiang et al., 2024
*NADPH oxidase inhibition*	ROS reduction	Oxidative ENS injury	Jiang et al., 2024
*Glutathione-mediated neuroprotection*	Antioxidant defense	Neurodegeneration, enteric neuropathies	Abdo et al., 2010
*BDNF signaling*	Anti-apoptotic and trophic support	ENS repair and regeneration	Steinkamp et al., 2012
*NGF signaling*	Neuronal survival and plasticity	ENS degeneration and recovery	von Boyen et al., 2006
*GDNF signaling*	Neuronal maintenance and regeneration	Neurodegenerative disorders	Hansebout et al., 2012
*Hormone-responsive EGC pathways*	Modulation of plasticity and neuroimmune signaling	Sex-specific therapeutic strategies	Emerging concept
*EGC-derived progenitor activation*	Endogenous neuronal replacement	Hirschsprung disease, ENS degeneration	Bondurand et al., 2003; Laddach et al., 2023; McCallum et al., 2020
*TACR3 / Neurokinin-B signaling*	Regulation of EGC diversification and functional states	Modulation of ENS plasticity and regeneration	Muppirala et al., 2026

Ultimately, a deeper understanding of how enteric glia integrate hormonal, inflammatory, and environmental signals may not only redefine current concepts of ENS biology but also uncover new strategies to promote regeneration and resilience within the GBA.
